# Trends and hotspots in gene research of epilepsy in children: A review and bibliometric analysis from 2010 to 2022

**DOI:** 10.1097/MD.0000000000034417

**Published:** 2023-07-28

**Authors:** Yuling Tian, Xilian Zhang, Hanjiang Chen, Caiyun Li, Liqing Niu, Qianfang Fu, Ping Rong, Rong Ma

**Affiliations:** a Department of Pediatrics, First Teaching Hospital of Tianjin University of Traditional Chinese Medicine, Tianjin, China; b National Clinical Research Center for Chinese Medicine Acupuncture and Moxibustion, Tianjin, China.

**Keywords:** bibliometrics, children, CiteSpace, epilepsy, gene, VOS viewer

## Abstract

About 70% to 80% of epilepsy cases are related to genetic factors. Genetic research has revealed the genetic etiology and molecular mechanisms of childhood epilepsy, which has increased our understanding of childhood epilepsy.

**Methods:**

We searched the core collection of Web of Science for relevant papers on genetic research on childhood epilepsy published since 2010 on November 30, 2022. In this study, original articles and reviews in English were included. Using CiteSpace and VOSviewer online tools, we conducted a bibliometric analysis of the countries, institutions, journals, co-cited journals, co-cited references, keywords, and research hotspots.

**Results:**

We evaluated 2500 literatures on epilepsy genomics in children. Among them, 96 countries published relevant articles, with the United States ranking the most. A total of 389 institutions have contributed relevant publications, and the University of Melbourne has published the most papers. Epilepsy journals were the most commonly cited. The references of papers were clustered into 9 categories: gene testing, epileptic encephalopathy, Dravet syndrome, focal cortical dysplasia, Rolandic epilepsy, copy number variation, ketogenic diet, monogenic epilepsy, and ptt2 mutation. Burst keywords represent the frontier of research, including developmental and epileptic encephalopathy (2021–2022), neurodevelopmental disorders (2020–2022), gene testing (2020–2022), and whole-exome sequencing (2019–2022).

**Conclusion:**

This study conducted a systematic and objective bibliometric analysis of the literature on epilepsy gene research in children. More importantly, it revealed the hot spot, frontier, and future developmental trends in the field. It will help pediatricians and geneticists further understand the dynamic evolution of genetic research on pediatric epilepsy.

## 1. Introduction

Epilepsy is a common brain disease that can cause serious physical damage to patients and greatly threaten their lifespan (the premature death rate of epilepsy patients is 2–3 times higher than that of the general population).^[1,2]^ According to statistics from the World Health Organization, more than 70 million people worldwide suffer from epilepsy, with an average of 1 person suffering from a lifelong illness every 7.60 people.^[3]^ The number of children with epilepsy worldwide is as high as 10.5 million, and 4 out of 5 of these children live in developing and economically underdeveloped countries.^[4]^ Among them, the incidence of epilepsy in children in China is 5–10%, and 400,000 new cases are reported annually.^[5]^

Epilepsy has strong genetic susceptibility,^[6]^ and genetic factors account for 70–80% of the incidence rate of epilepsy.^[7]^ Approximately 30% of genetic susceptibility to systemic epilepsy can be explained by common genetic variation.^[8]^ In contrast, as a first-line treatment method, antiepileptic drugs mainly focus on regulating the imbalance of neuronal excitation and inhibition but cannot affect or reverse the potential progression of the disease^[9]^; almost one-third of epileptic children have no effect on drug treatment.^[10]^ Non-pharmacotherapies, such as surgery and ketogenic diet, have certain limitations in their applicability to the population.

Fortunately, with the continuous progress in genomics and molecular biology technology in recent years, research on the pathophysiological mechanisms of epilepsy has been greatly promoted. An increasing number of pathogenic genes related to epilepsy have been discovered, including ion transport, cell growth and differentiation, synaptic process regulation, intracellular and intercellular small-molecule transport and metabolism, and gene transcription and translation regulation, among which ion channel genes are the most common.^[11]^ Zhang proposed that gene therapy may be a promising alternative to traditional pharmacological tools and surgical treatments for epilepsy.^[9]^ The main approach is gene transmission mediated by adeno-associated viruses encoding neuroregulatory peptides, neurotrophins, enzymes, and potassium channels.^[12]^ From the above, it can be seen that genetic research has accelerated the understanding of the genetic causes of childhood epilepsy, while also providing accurate treatment and a more accurate understanding of prognosis.^[13]^ Therefore, considering the diversity of epileptic factors, individualized treatment for each child with epilepsy is important.^[14]^ It can even be said that in the future, all epilepsy diseases may need to consider the risk of genetic mutations.^[15,16]^

Bibliometric analysis is a research method based on bibliometrics and takes literature data as the research object, which has strong data analysis and visualization capabilities.^[17]^ It can not only be used for qualitative and quantitative analysis of a discipline, including authors, institutions, countries, and regions, as well as co-cited authors, journals, and references, but can also help researchers quickly present research hotspots and development trends in childhood epilepsy gene research.^[18]^ Compared to traditional reviews, bibliometric analysis has the advantage of being more intuitive and focused.

Currently, there have been some metrology studies on epilepsy and its complications, including epilepsy with autism^[19]^ and epilepsy with Alzheimer disease.^[20]^ In addition, bibliometric analysis is used to analyze epilepsy during special periods such as gestational epilepsy.^[21]^ To date, there has been a metrology analysis of relevant literature on epilepsy genomics,^[22]^ but there is a lack of detailed literature on children’s epilepsy genomics, which has been proven to be important by an increasing number of studies and evidence.

Therefore, this study aimed to delve into the genetic research of childhood epilepsy and evaluate the current research status and hotspots in this field. This study is expected to provide a reference for future research by scholars and researchers in the fields of childhood epilepsy and genomics.

## 2. Materials and methods

**Figure 1.**
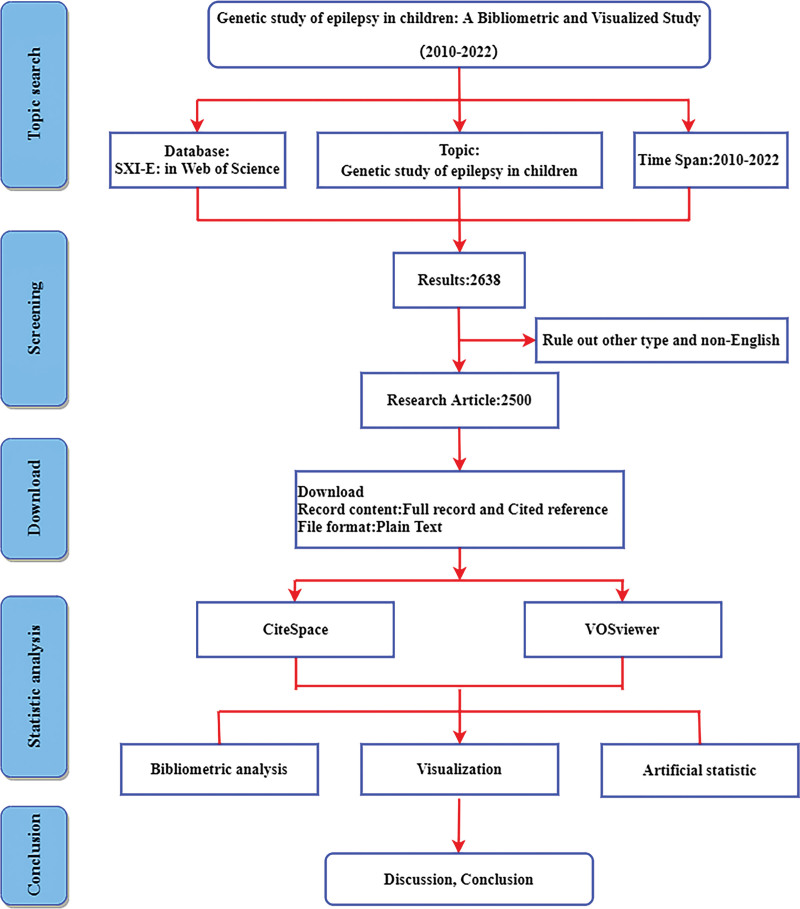
Flow chart of document retrieval and analysis.

All relevant publications were obtained from the core collection of the Web of Science. The retrieval strategy was TS = (gene OR geno *) AND TS = (epileps * OR “size order *” OR aura *) AND TS = (child *). The document type is article or comment. The language type is English and the time span is from 2010 to 2022. Data extraction completed all included studies on November 30, 2022. After retrieval and deduplication, 2500 documents were finally included; 2 authors independently verified all the data, 1 (XZ) retrieved the data, and the other rechecked the data (YT). The detailed search and analysis processes are shown in Figure 1.

Software VOSviewer 1.6.18 and CiteSpace 5.8. R3 is conducted for bibliometric analysis and visual network atlas analysis of retrieved data.^[21,23]^ We describe the characteristics of the publications, including journals and co-cited journals, countries or regions, institutions, references, and keywords. These include valuable parameters, such as the number of documents, sudden keywords, and centrality. The number of citations represents how influential and important the literature is in the field.^[24]^ Burst keywords were cited on the research frontier.^[25]^ Nodes with high centrality (≥0.1) are highlighted with red rings in the network and are generally considered influential in the high network.^[26]^

Through the above analysis, the current characteristics, discipline development track, research hotspots, and future research directions in the application of genetics to childhood epilepsy can be obtained.

## 3. Results

### 3.1. Annual Distribution of Publications

**Figure 2.**
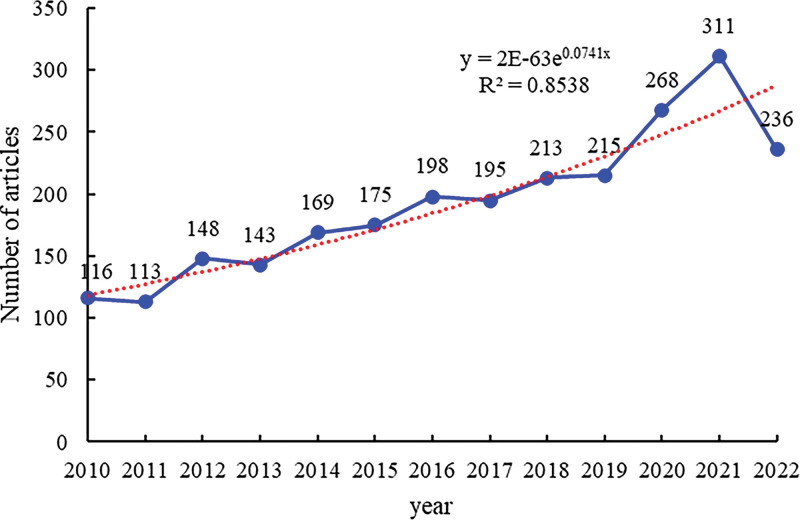
Trend chart of annual document volume.

A total of 2500 articles were published between 2010 and 2022, as shown in Figure 2. The dots indicated by the circle on the blue line indicate the number of articles published each year. The red dotted line indicates the overall trend of the number of documents issued. The closer the formula R^2^ = 0.8538 is to 1, the stronger the authenticity. Since 2010, it has fluctuated and has shown a rapid growth trend since 2019. Because the data for 2022 is slightly lower than that for 2021, this cannot change the overall growth trend. Therefore, under this general trend, it is expected that the genetic research field of childhood epilepsy will remain the focus of scholars for the next few years.

### 3.2. Analysis of countries/regions and institutions

**Figure 3.**
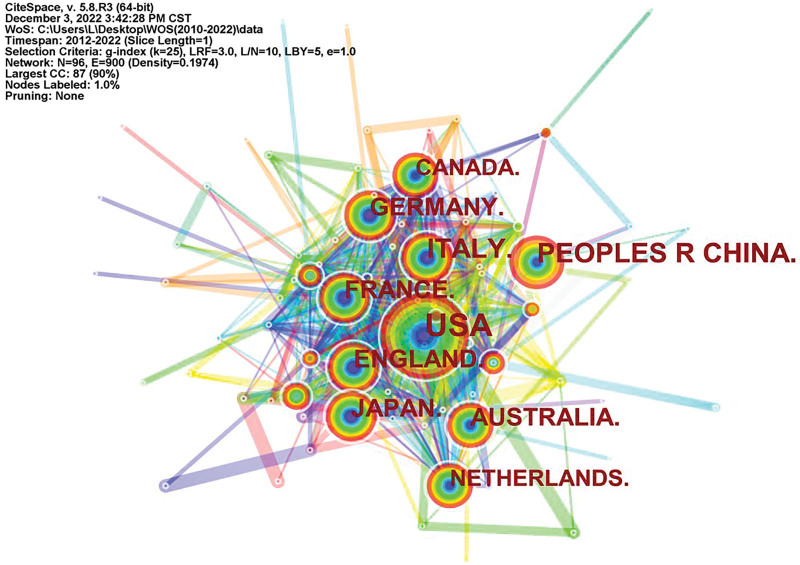
Diagram of national cooperation.

Between 2010 and 2022, 96 countries/regions published relevant articles (Fig. 3). After the centrality of nodes is calculated, we can get the nodes with strong influence in the network map through the numerical value. Nodes with red circles are considered to have a sudden increase in the number of publications in a short period of time, that is, nodes with greater impact on the network. Table 1 shows the centrality values and count of each country in the top 10. France has the advantage of the highest centrality (0.20), and Germany and the United Kingdom are both ranked second, with a centrality of 0.09. On the other hand, in terms of the number of publications, the results show that the United States is the leading country (682), followed by China (354), Italy (308), and Germany (227). Based on the above data, it can be concluded that the United States and the People’s Republic of China play a major role in the collaboration network among all countries.

**Table 1:** Top 10 countries and institutions related to gene research of childhood epilepsy.

Rank	Country	Count	Centrality	Institution	Count	Centrality
1	USA	682	0.02	Univ Melbourne	88	0.08
2	PEOPLES R CHINA	354	0.01	Boston Childrens Hosp	53	0.14
3	ITALY	308	0.01	Harvard Med Sch	53	0.02
4	GERMANY	227	0.09	Univ Washington	50	0.03
5	ENGLAND	204	0.09	Univ Calif San Francisco	45	0.02
6	AUSTRALIA	163	0.07	Childrens Hosp Philadelphia	45	0.07
7	FRANCE	163	0.2	Columbia Univ	42	0.04
8	JAPAN	160	0.05	Baylor Coll Med	41	0.08
9	CANADA	143	0.08	Univ Genoa	37	0.05
10	NETHERLANDS	124	0.07	UCL	36	0.06

**Figure 4.**
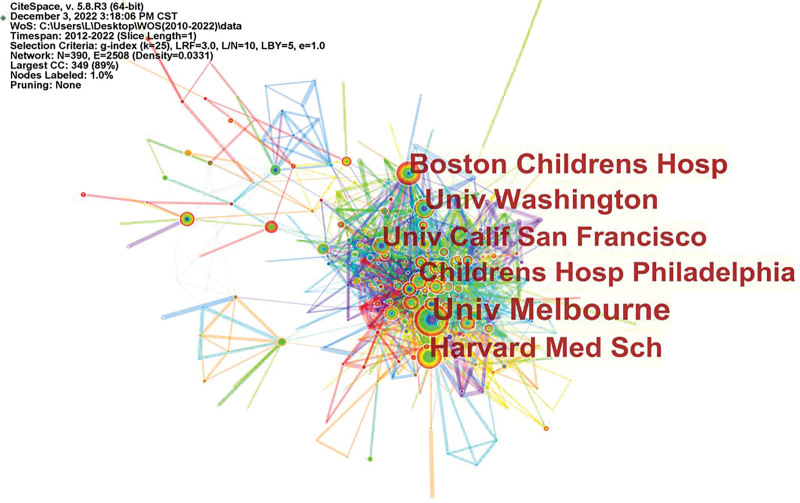
Diagram of institutional cooperation.

In total, 389 institutions published relevant articles (Fig. 4). Among the top 10 major international institutions with contributions (Table 1), the University of Melbourne is the leading institution in terms of the number of publications (n = 88), followed by Boston Children’s Hospital (n = 53), Harvard Medical School (n = 53), and the University of Washington (n = 50). Boston Children’s Hospital is the only institution with centrality ≥ 0.1, which shows that Boston Children’s Hospital plays an important role in this field (Fig. 4).

### 3.3. Journals analysis

**Figure 5.**
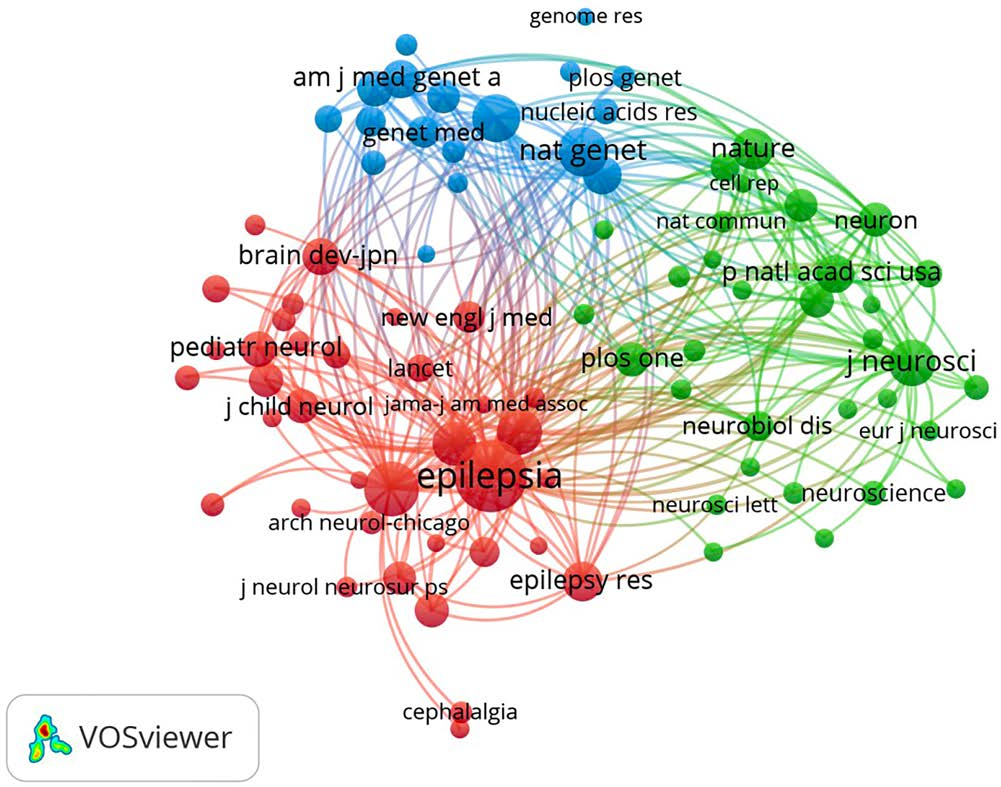
Network of cited journals.

The top 10 journals were frequently cited (Table 2). EPILEPSY has the most citations among all the cited journals (citations = 1427, IF = 6.740), followed by NEUROLOGY (citations = 1228, IF = 11.800), and AM J HUM GENET (citations = 1063, IF = 11.043). Among these journals, NAT GENET has the highest impact factor (citations = 993, IF = 41.307). Among the top 10 journals, 8 were in the Q1 area of the JCR classification. Figure 5 shows the cooperation between the cited journals.

**Table 2:** Top 10 co-cited journals related to gene research of childhood epilepsy.

Rank	Journal title	IF (2021)	JCR division	Total number of citations
1	EPILEPSIA	6.740	Q1	1427
2	NEUROLOGY	11.800	Q1	1228
3	AM J HUM GENET	11.043	Q1	1063
4	NAT GENET	41.307	Q1	993
5	BRAIN	15.255	Q1	929
6	ANN NEUROL	11.274	Q1	909
7	NATURE	69.504	Q1	757
8	EPILEPSY RES	2.991	Q3	731
9	J MED GENET	5.941	Q1	686
10	PLOS ONE	3.752	Q2	684

**Figure 6.**
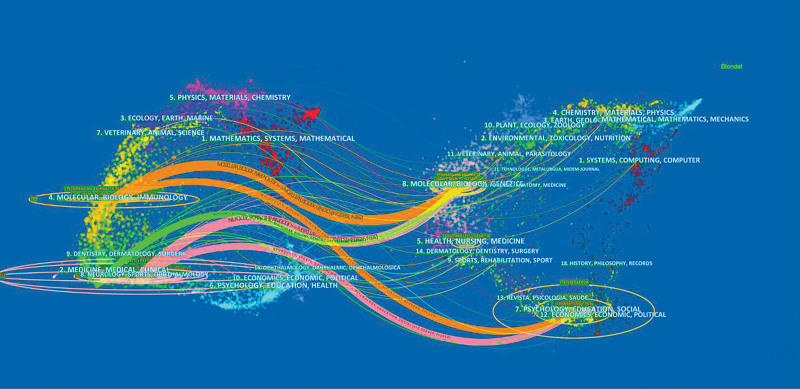
Double overlay of periodicals.

The relationship between references shows the knowledge exchange between research fields, which summarizes the knowledge base and research status, and the cited articles constitute the current research direction and the knowledge frontier.^[27]^ The double-map coverage of journals represents the subject distribution of the academic journals (Fig. 6). In the picture, the left side is the citing journal and the right side is the co-cited journal. The citation relationship between them is represented by the colored path in the middle.

There are 5 reference paths. The first and second orange paths are articles published in molecular/biological/genetic journals and articles published in psychological/educational/social journals, which are primarily cited by researchers published in molecular/biological/immunological journals; the third green path is articles published in molecular/biological/genetic journals, primarily cited by research published in drug/medical/clinical journals; and the fourth and fifth pink paths are articles published in molecular/biological/genetic journals and in psychological/educational/social journals, which are mainly cited by research published in neurology/sports/ophthalmology.

### 3.4. Reference analysis

References are the key indicators of literature measurement because the frequently cited literature has a significant impact on its research field. As shown in Table 3, this article, published in EPILEPSY, has been cited 128 times, ranking first. We summarized the subject of highly cited references, and the results showed that the terminology, classification, and gene sequencing standards of epilepsy were standardized. Moreover, multiple gene mutations such as EGRIN2A, CHD2, and SYNGAP1 were found to be the main causes of epileptic encephalopathy (EE). These results have greatly improved genomic research on epilepsy in children. The greater the centrality of these references, the more important are the documents (Table 2).

**Table 3:** Top 10 references related to gene research of epilepsy in children.

Rank	Source titles	DOI	Title of cited reference	Count	Centrality	Interpretation of the findings
1	EPILEPSIA	10.1111/epi.13709	ILAE classification of the epilepsies: Position paper of the ILAE Commission for Classification and Terminology	128	0.02	This paper updated the classification of epilepsy and introduced some new terms
2	GENET MED	10.1038/gim.2015.30	Standards and guidelines for the interpretation of sequence variants: a joint consensus recommendation of the American College of Medical Genetics and Genomics and the Association for Molecular Pathology	122	0	This paper reports the standards for interpretation of sequence variants in gene sequencing
3	NATURE	10.1038/nature12439	De novo mutations in epileptic encephalopathies	97	0.07	This paper detected 329 novel mutation genes of epileptic encephalopathy by gene sequencing
4	EPILEPSIA	10.1111/j.1528-1167.2010.02522.x	Revised terminology and concepts for organization of seizures and epilepsies: report of the ILAE Commission on Classification and Terminology, 2005–2009	64	0	This article revised and reported the terms and concepts of epilepsy
5	NAT GENET	10.1038/ng.2646	Targeted resequencing in epileptic encephalopathies identifies de novo mutations in CHD2 and SYNGAP1	62	0.07	In this paper found that CHD2 and SYNGAP1 mutations are the cause of epileptic encephalopathy by directional large-scale parallel re sequencing
6	NATURE	10.1038/nature19057	Analysis of protein-coding genetic variation in 60,706 humans	60	0.02	This paper proposes that the reference data set of human genetic variation can be used to effectively filter candidate pathogenic variants
7	LANCET NEUROL	10.1016/S1474-4422(15)00250-1	The genetic landscape of the epileptic encephalopathies of infancy and childhood	50	0.02	This article summarized the genetic content of epileptic encephalopathy
8	NAT GENET	10.1038/ng.2727	GRIN2A mutations cause epilepsy-aphasia spectrum disorders	44	0.03	This article found that EGRIN2A mutation was limited to epileptic aphasia syndrome
9	NAT GENET	10.1038/ng.2726	GRIN2A mutations in acquired epileptic aphasia and related childhood focal epilepsies and encephalopathies with speech and language dysfunction	40	0.02	This article believes that GRIN2A is the main gene that causes epileptic encephalopathy
10	NAT GENET	10.1038/ng.2728	Mutations in GRIN2A cause idiopathic focal epilepsy with rolandic spikes	38	0.02	This study confirmed that the change of gene encoding NR2A subunit of NMDA receptor is the main genetic risk factor of IFE

ILAE = International League Against Epilepsy, IFE = idiopathic focal epilepsy.

**Figure 7.**
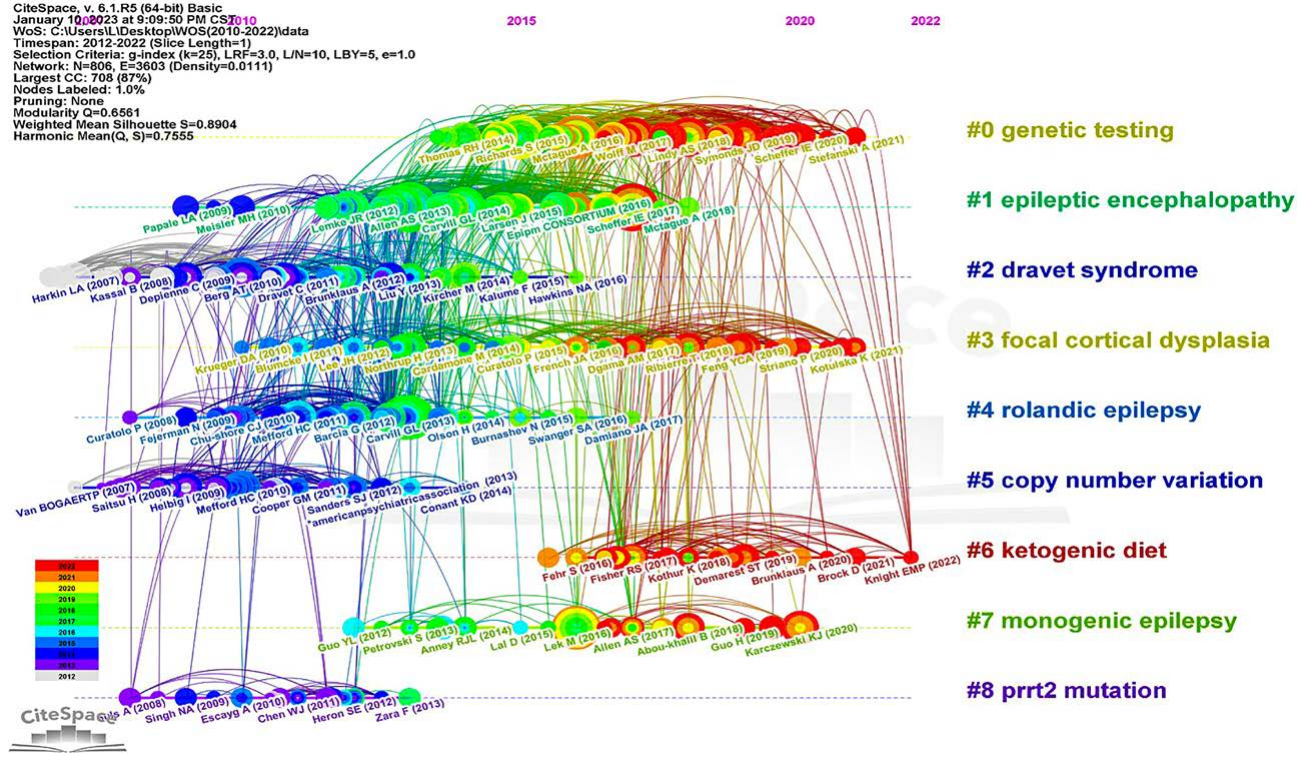
Reference co-citation map of publications.

In this study, based on cluster analysis of co-cited references, we constructed a timeline chart (Fig. 7) to test the scientific correlation of relevant articles. The timeline diagram clearly shows the distribution of references at different times, and the size of nodes on the horizontal line represents the influence of the citation. Meanwhile, index items are used as cluster tags (# 0– # 8); The largest cluster # 0 is “gene testing,” # 1 is “epileptic encephalopathy,” # 2 is “dravet syndrome,” # 3 is “focal cortical dysplasia,” # 4 is “rolandic epilepsy,” # 5 is “copy number variation,” # 6 is “ketogenic diet,” # 7 is “single gene epilepsy,” and # 8 is “ptt2 mutation.”

### 3.5. Keyword analysis

**Figure 8.**
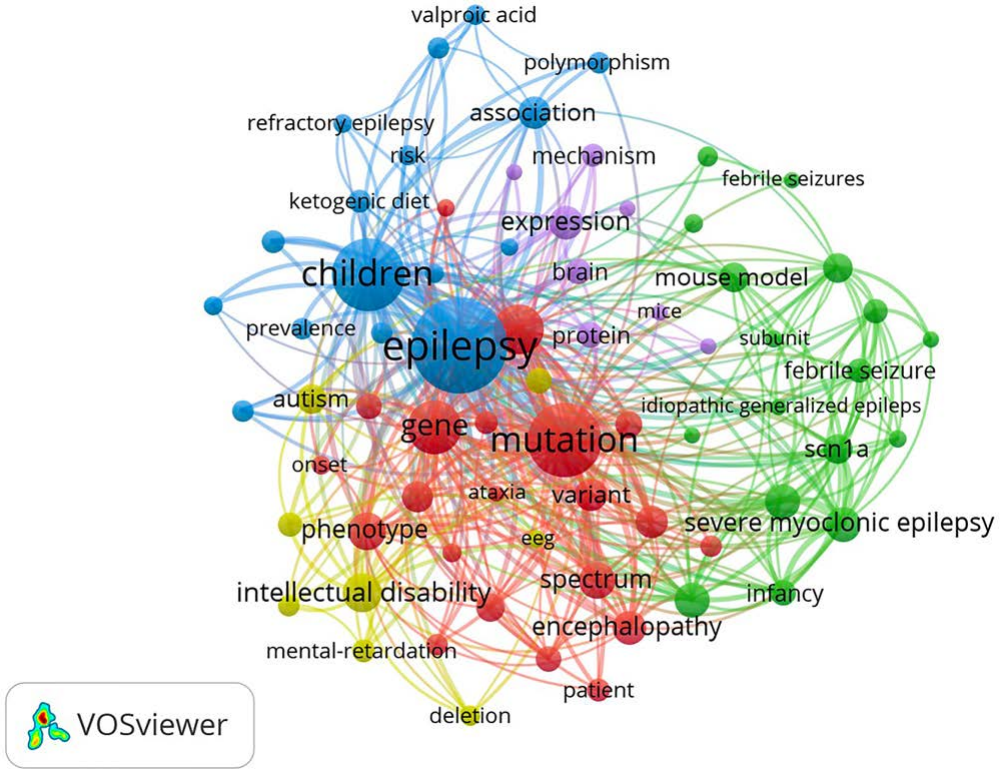
Network map of keywords.

**Figure 9.**
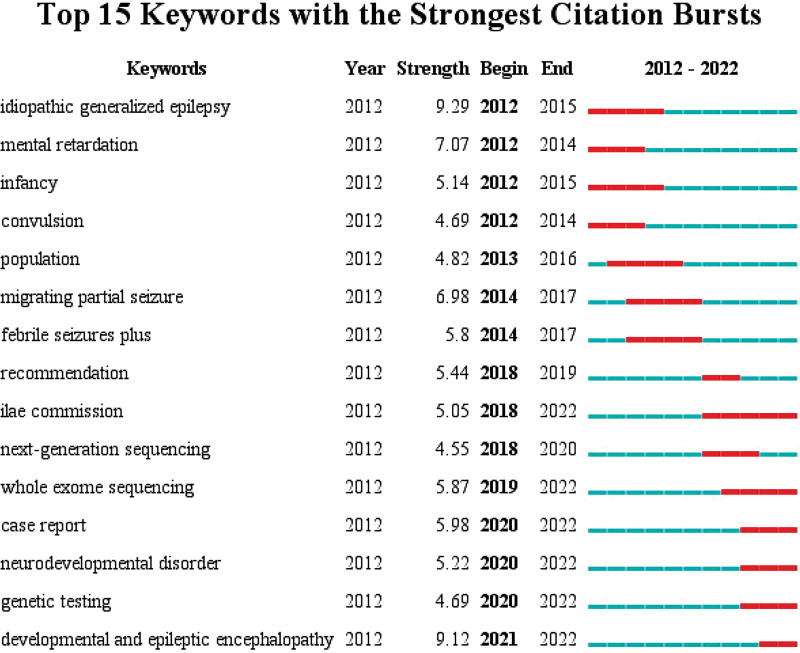
Keywords with the strongest citation bursts of publications.

The co-occurrence of keywords in relevant publications was extracted and analyzed to explore the key content of genetic study of epilepsy in children. Table 4 shows the top 20 keywords highly related to co-occurrence frequency. When determining the theme of the research field, we analyzed the keywords in 2500 documents, and finally determined 70 keywords, of which each keyword appeared at least 40 times (Fig. 8). Using the number of occurrences as the threshold, a co-occurrence network diagram based on keywords was constructed, as shown in Figure 8. There were 5 clusters of different colors. The size of the circle has a positive correlation with the co-occurrence of keywords, and keywords of the same color together show stronger links. Epilepsy, mutation, and children constituted the largest circle among all keywords determined by co-occurrence analysis. Our research also investigated the time trend of hotspot transfer by quoting the top 15 keywords with the strongest outbreak. These predictions include developmental and epileptic encephalopathy (DEE) (2021–2022), neurodevelopmental disorders (2020–2022), gene testing (2020–2022), and whole-exome sequencing (2019–2022) (Fig. 9).

**Table 4:** Top 20 related to gene research of epilepsy in children.

Rank	Keyword	Occurrence	Link strength	Rank	Keyword	Occurrence	Link strength
1	Epilepsy	954	3044	11	Dravet syndrome	157	722
2	Mutation	603	2008	12	Expression	154	492
3	Children	587	1941	13	Encephalopathy	154	605
4	Gene	343	1238	14	Genetics	139	553
5	Seizure	305	1209	15	Variant	138	558
6	Intellectual disability	198	692	16	Association	137	486
7	Phenotype	180	728	17	Disorder	130	473
8	Spectrum	171	649	18	Mouse model	123	450
9	Severe myoclonic epilepsy	164	808	19	Scn1a	122	638
10	De novo mutation	161	652	20	Childhood absence epilepsy	121	439

## 4. Discussion

### 4.1. General data

This study analyzed 2500 SCI papers published from 2010 to 2022 related to the study of epilepsy genes in children. Since 2019, the number of published articles has rapidly increased with the development of gene sequencing technology. This shows that the development of epilepsy gene research in children has attracted widespread attention. The United States ranked first (682), accounting for 27.3%. China’s publications ranked second (354), accounting for 14.2% of the total publications. The top ten institutions are from developed countries, including 7 in the United States, one in Australia, one in Italy, and one in the United Kingdom. EPILEPSY was the most frequently published journal. These observations indicate that the journal EPILEPSY has made major contributions to research in this field. In addition, we surveyed the top 10 papers cited. The first article was published on EPILEPSY by the International League Against Epilepsy Commission and was cited 128 times. The second article was published by Richards on GENET MED and was cited 122 times. Highly cited publications will provide insight for researchers in this field.

### 4.2. Knowledge Base

In previous studies, the application of childhood epilepsy research in genetics has yielded significant results. As shown in Fig. 6, after the cluster was co-referenced, the key cluster nodes successfully identified the knowledge base, namely: # 0 is “genetic testing,” # 1 was “epileptic encephalopathy,” # 2 was “dravet syndrome,” # 3 is “focal cortical dysplasia,” # 4 is “rolandic epilepsy,” # 5 is “copy number variation,” # 6 is “ketogenic diet,” # 7 is “monogenic epilepsy,” and # 8 is “ptt2 mutation.” We divided the cluster sets into 3 categories to describe the knowledge base (Table 5).

**Table 5:** Cluster classification of references.

Category name	Cluster label	Size	Silhouette	Mean (year)
Disease	#1 epileptic encephalopathy	108	0.842	2014
	#2 dravet syndrome	106	0.926	2010
	#3 focal cortical dysplasia	73	0.946	2016
	#4 rolandic epilepsy	69	0.816	2012
	#7 monogenic epilepsy	25	0.934	2016
Gene detection and mutation	#0 genetic testing	151	0.821	2017
	#5 copy number variation	54	0.908	2010
	#8 ptt2 mutation	23	0.987	2011
Treatment	#6 ketogenic diet	30	0.962	2018

### 4.3. Disease

The disease knowledge base includes 5 clusters, namely: # 1 “epileptic encephalopathy,” # 2 “dravet syndrome,” # 3 “focal cortical dysplasia,” # 4 “rolandic epilepsy,” and # 7 “monogenic epilepsy.” From the above clusters, the key diseases in the study of epilepsy genetics in children can be identified.

In the # 1 “epileptic encephalopathy” cluster, EE usually includes refractory epilepsy, various types of motor disorders, and different degrees of developmental retardation.^[28]^ The diagnosis rate of younger patients, especially those with seizures during the neonatal period, is higher in patients with drug-resistant epilepsy.^[29]^ In recent years, many new mutations have been identified in individuals with epilepsy encephalopathy, including SCN8A, KCNT1, and other gene mutations.^[30,31]^

In the # 2 “ Dravet syndrome” cluster, we learned about the clinical characteristics, diagnosis, and treatment status of the Dravet syndrome. Dravet syndrome is one of the most serious epilepsy syndromes in early childhood and is usually characterized by febrile epileptic status with a high childhood mortality rate (up to 15%).^[32]^ In 70–80% of individuals with Dravet syndrome, its pathogenesis is related to a mutation in the sodium channel alpha 1 subunit gene (SCN1A).^[33]^

In the # 3 “focal cortical dysplasia” cluster, many studies have proved that focal cortical dysplasia (FCD) is the most common cause of drug-resistant focal epilepsy in children and young adults. The diagnosis of the currently defined FCD subtype depends on histopathological evaluation of the surgical brain tissue.^[34]^ Surgery is the primary treatment option for FCD-related epilepsy. In recent years, advanced neurophysiological and neuroimaging technologies have improved surgical results and reduced the risk of postoperative defects.^[35]^

In the # 4 “rolandic epilepsy” cluster, rolandic epilepsy (RE) is the most common focal epilepsy in childhood and its important genetic risk factors have been identified. Dimassi et al detected 30 rare copy number variants by array comparative genomic hybridization and quantitative polymerase chain reaction analysis, including BRWD3, GRIN2A, KCNC3, PRKCE, PRRT2, SHANK1, and TSPAN7.^[36]^ Reinthaler et al demonstrated that 16p11.2 duplication is an important genetic risk factor for typical and atypical RE.^[37]^

In the # 7 “monogenic epilepsy” cluster, the cause of the monogenic mutation of epilepsy, mainly caused by Dravet syndrome, has been reported. Zou et al found that the most common epilepsy-related duplication copy number variations were 17p13.3, 16p11.2, and 7q11.23.^[38]^ Nappi et al found that mutations in KCNQ2, KCNQ3, and KCNQ5 were associated with DEE.^[39]^

### 4.4. Gene detection and mutation

This knowledge base includes # 0 “genetic testing,” # 5 “copy number variation, “ and # 8 “ptt2 mutation.”

In the # 0 “genetic testing” cluster, the application of gene testing can greatly assist children with epilepsy in identifying genetic causes and in accurate diagnosis and treatment. Bayat et al applied gene detection to 118 people and carried out gene diagnosis for approximately 50% of the patients with non-acquired epilepsy, enabling them to adopt precise treatment, thus reducing the burden of epilepsy in these patients.^[40]^ Palmer et al believe that advanced therapies, such as genome sequencing technology and gene therapy, provide unprecedented precision medical opportunities for parents of children with severe but rare neurological diseases.^[41]^ Smith et al strongly recommended genetic testing for all patients with unexplained epilepsy.^[42]^

In the # 5 “copy number variation” cluster, it was discussed that partial deoxyribonucleic acid fragment variation is the genetic cause of epilepsy or epilepsy-comorbid autism. Wegiel et al have shown that chromosome 15q11.2-13.1 duplication syndrome has more serious focal developmental defects, which may lead to early onset of refractory epilepsy and sudden death from epilepsy.^[43]^ Orosco et al found that deletion of the Wdfy3 gene resulted in migration defects in cortical projection neurons, leading to a significant co-disease of epilepsy and autism.^[44]^

In the # 8 “ptt2 mutation” cluster, we learned that PRRT2 is the most common single-gene epilepsy and common epilepsy type. Usluer et al found that the PRRT2 mutation is the most common cause of benign familial infantile epilepsy and is found in approximately 80% of benign familial infantile epilepsy families.^[45]^ Okumura et al found that PRRT2 mutations are very common in Japanese patients with benign infantile epilepsy, especially in those with a family history of paroxysmal dyskinesia.^[46]^

### 4.5. Treatment

In previous studies in the field of epilepsy genetics in children, only # 6 “ketogenic diet” was involved in the cluster set of treatment. In the # 6 “ketogenic diet” cluster, in recent years, many studies have proved that ketogenic diet is helpful for the treatment of hereditary epilepsy and drug-resistant epilepsy. This is especially true for CDKL5 deficiency in patients with developmental epilepsy encephalopathy.^[47]^ Other studies have shown that ketogenic diet can change intestinal microorganisms during the treatment of drug-resistant epilepsy, thereby regulating the immune system and improving inflammation, revealing the potential mechanism of ketogenic diet in the treatment of epilepsy.^[48]^

Summing up the above clusters suggests that the field of epilepsy genetics in children is more inclined toward gene diagnosis, at present, while the corresponding targeted and precise treatment is not mature, and further exploration and strengthening is urgently needed.

### 4.6. Hot spots and frontiers

Keywords focus on the key issues of current research, while burst keywords forecast the frontiers and possible hot spots of future research. In our study, we identified 4 keywords with the strongest citation burst, including DEE (2021–2022), neurodevelopmental disorders (2020–2022), gene testing (2020–2022), and whole-exome sequencing (2019–2022). These keywords represent research frontiers and future directions of gene research on epilepsy in children.

### 4.7. Developmental and epileptic encephalopathy (2021–2022)

DEE is a group of diseases characterized by the coexistence of epilepsy and intellectual disability, in which there are additional developmental disorders independent of epileptic activity. It is associated with a high mortality and incidence rate. With the development of gene sequencing, more than 400 genes have been found to be associated with DEE, and genetic causes have been identified in more than 50% of patients have found genetic causes.^[49]^ In recent years, genetic research on epilepsy in children has focused on the development of specific genetic DEE phenotypes to facilitate early diagnosis and better treatment targets. At present, we treat patients with many genetic causes of DEE in infancy, including genes encoding sodium or potassium channel subunits, tuberous sclerosis, and congenital metabolic diseases.^[50]^ In addition, Nappi et al revealed that mutations in KCNQ2, KCNQ3, and more rare KCNQ5 genes are related to DEE and described the clinical characteristics and pathogenesis of DEE patients with new KCNQ5 variants.^[39]^

### 4.8. Neurodevelopmental disorders (2020–2022)

Neurodevelopmental disorder (NDD) is closely related to epilepsy, and has been a hot research topic in epilepsy genetics in recent years. Poeta et al observed a disorder in the transcription pathway in various forms of NDD, such as intellectual disability, epilepsy, and autism spectrum disorder, which revealed a new insight into the epigenetic process supporting the pathogenesis of NDD and provided a new way to evaluate the development opportunity and the key window of potential treatment.^[51]^ Alehabib et al showed that approximately 90% of epilepsy patients had different types of NDDs in childhood and adolescence, such as intellectual disability, developmental stagnation, and behavioral disorders.^[52]^ Mellone et al applied targeted next-generation sequencing technology and found that the diagnostic rate of NDD in women was significantly higher than that in men, especially for autism spectrum disorder and epilepsy.^[53]^

### 4.9. Genetic testing (2020–2022)

Genetic testing has become a routine part of the diagnosis of children with early epilepsy and plays a key role in the diagnosis of most developmental epilepsy encephalopathies and several idiopathic epilepsies. Striano, PP, and others summarized several available gene tests, including targeted detection and revolutionary tools, which make it possible to sequence all coding (whole exon) and noncoding (whole genome) regions of the human genome.^[54]^ Lee et al used retrospective observational research to explore the diagnostic rate and practicality of gene detection. The results showed that the diagnostic rate of WES was the highest, followed by microarray, single-gene test, and targeted polygenic group test.^[55]^ It is strongly recommended to carry out gene detection for all patients with unexplained epilepsy in the order of exon/genome sequencing and/or multiple genomes (>25 genes), followed by chromosome microarray detection.^[42]^

### 4.10. Whole-exome sequencing (2019–2022)

In recent years, WES has become a powerful tool to reveal the genetic heterogeneity of epilepsy in children. The application of these tests in children and adults with epilepsy has led to the identification of new pathogenic genes, broadened our knowledge of epilepsy pathophysiology, and produced therapeutic significance.^[56]^ Sun et al evaluated the possibility of analyzing single nucleotide variations, indels, and copy number variations that lead to diseases in a single test based on WES in EEs and found that WES can significantly improve the diagnostic rate of EE, and confirmed 2 new pathogenic genes, CACNA1E and WDR26.^[57]^ Salinas et al reported the diagnostic utility of targeted genome sequencing and WES in 55 patients with DEE; that is, the overall diagnostic rate increased to 53% and new variants in genes (CHD2, COL4A1, FOXG1, etc.) were identified, which strongly demonstrated the importance of reevaluating gene testing in subjects without determining the cause.^[58]^

## 5. Conclusion

This study used bibliometrics to systematically and objectively analyze the relevant literature on the etiology of childhood epilepsy. Importantly, we identified the research basis, current hotspots, and future trends in the field of genetics in childhood epilepsy. These knowledge bases include “genetic testing,” “epileptic encephalopathy,” “dravet syndrome,” “focal cortical dysplasia,” “rolandic epilepsy,” “copy number variation,” “ketogenic diet,” “monogenic epilepsy, and “ptt2 mutations “. We also provide hotspots and cutting-edge guidance for scholars who wish to conduct research in this field in the future. More importantly, emerging trends were identified, including DEE, genetic testing, neurodevelopmental disorders, and WES. Finally, the literature selected in this study is not comprehensive, which may lead to publication bias that will affect the results of this review to a certain extent.

## 6. Limitation

This is the first time to use CiteSpace and VOSviewer to explore the genetic research of epilepsy in children. However, our study has some limitations. First, this study did not include all the relevant literature. Some noncore collections and non-article or review types of research were excluded. Second, we only analyzed research in the English language, and the results of this study may not be applicable to research published in other languages. Third, due to the low number of citations in recently published research, there are differences between the results of bibliometric analysis and actual research.

## Author contributions

**Conceptualization:** Yuling Tian, Xilian Zhang, Hanjiang Chen, Rong Ma.

**Data curation:** Caiyun Li, Qianfang Fu, Ping Rong.

**Funding acquisition:** Xilian Zhang, Hanjiang Chen.

**Methodology:** Yuling Tian, Caiyun Li, Liqing Niu, Qianfang Fu.

**Resources:** Liqing Niu.

**Software:** Liqing Niu, Qianfang Fu.

**Supervision:** Hanjiang Chen, Ping Rong, Rong Ma.

**Visualization:** Yuling Tian, Caiyun Li, Liqing Niu, Ping Rong.

**Writing – original draft:** Yuling Tian, Xilian Zhang.

**Writing – review & editing:** Yuling Tian, Xilian Zhang, Hanjiang Chen, Rong Ma.
